# Supplementary material for: Complete chemical structures of human mitochondrial tRNAs
Source: Nat Commun. 2020 Aug 28;11:4269. doi: 10.1038/s41467-020-18068-6 (PMC7455718; doi:10.1038/s41467-020-18068-6)
Supplement: Supplementary file 1 — Supplementary Information [file 41467_2020_18068_MOESM1_ESM.pdf]

Supplementary information

Complete chemical structures of human mitochondrial tRNAs

Takeo Suzuki et al.

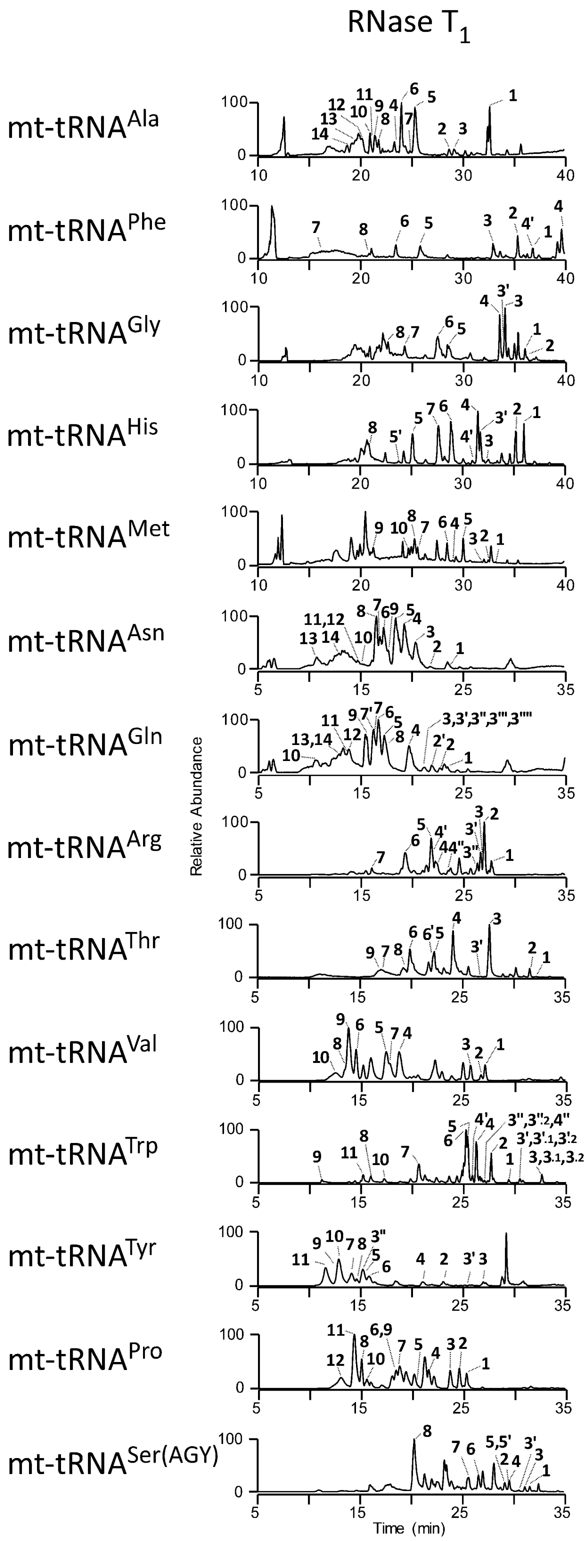

Supplementary Figure 1. RNA fragment analysis of human mt-tRNAs.

Base peak chromatogram (BPC) for each tRNA digested by RNase T$_1$ (left panels) and RNase A (right panels). All assigned fragments (numbered) listed in Supplementary Data 1.

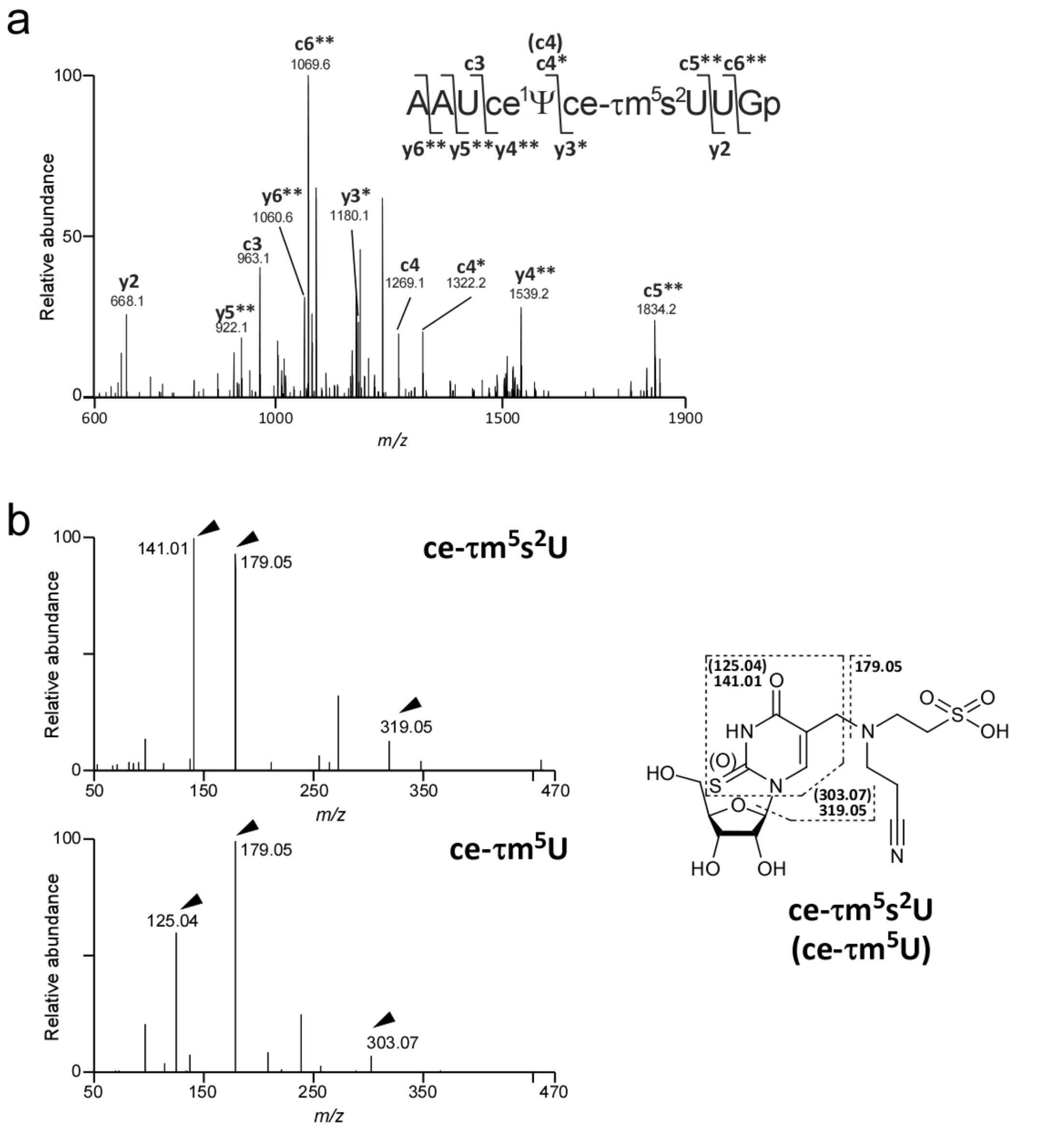

Supplementary Figure 2. Cyanoethylation of τm^{5}s^{2}U34

(a) CID spectrum of a di-cyanoethylated fragment from human mt-tRNA^{Gln}. The doubly charged negative ion of the RNA fragment (*m/z* 1251.1) was used as a precursor for CID. Assignment of the product ions revealed two cyanoethylation sites at Ψ33 and τm^{5}s^{2}U34. Asterisks marked on the product ions indicate the number of cyanoethylations.

(b) CID spectra (left panels) of the cyanoethylated τm^{5}s^{2}U and τm^{5}U nucleosides [ce-τm^5(s^2)U] indicate that cyanoethylation occurs at the N atom in the taurine moiety. The predicted chemical structure of the derivative and its dissociation patterns, with *m/z* values of the product ions, are shown on the right. Assigned product ions are indicated by arrowheads in the CID spectra.

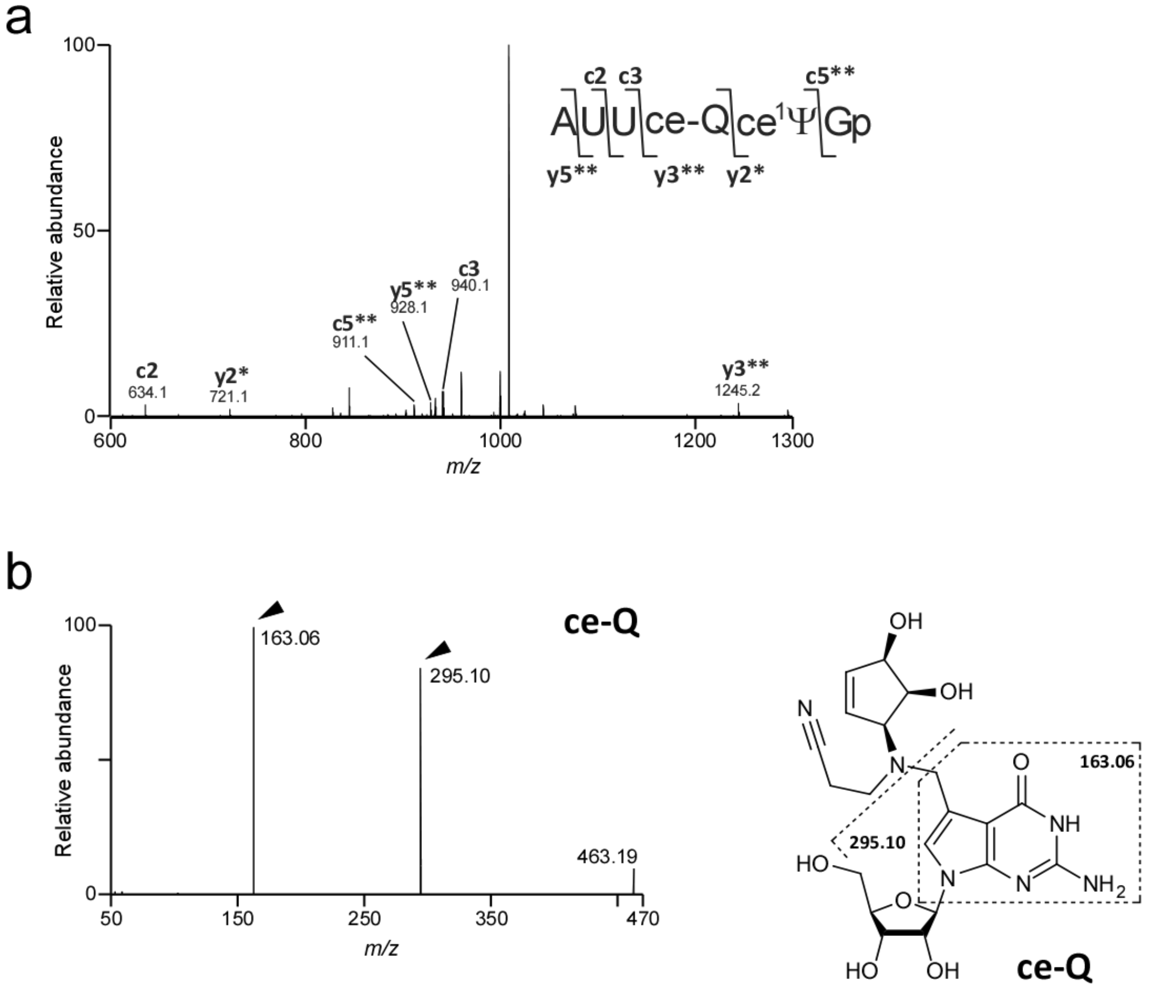

Supplementary Figure 3. Cyanoethylation of Q34

(a) CID spectrum of a di-cyanoethylated fragment from human mt-tRNA^{His}. The doubly charged negative ion of the RNA fragment (*m/z* 1092.7) was used as a precursor for CID. Assignment of the product ions revealed two cyanoethylation sites at Q34 and Ψ35. Asterisks marked on the product ions indicate the number of cyanoethylations.
(b) CID spectrum (left panel) of the cyanoethylated Q34 nucleosides (ce-Q) indicates that cyanoethylation occurs at the N atom in the side chain. The predicted chemical structure of the derivative and its dissociation patterns, with *m/z* values of the product ions, are shown on the right. Assigned product ions are indicated by arrowheads in the CID spectrum.

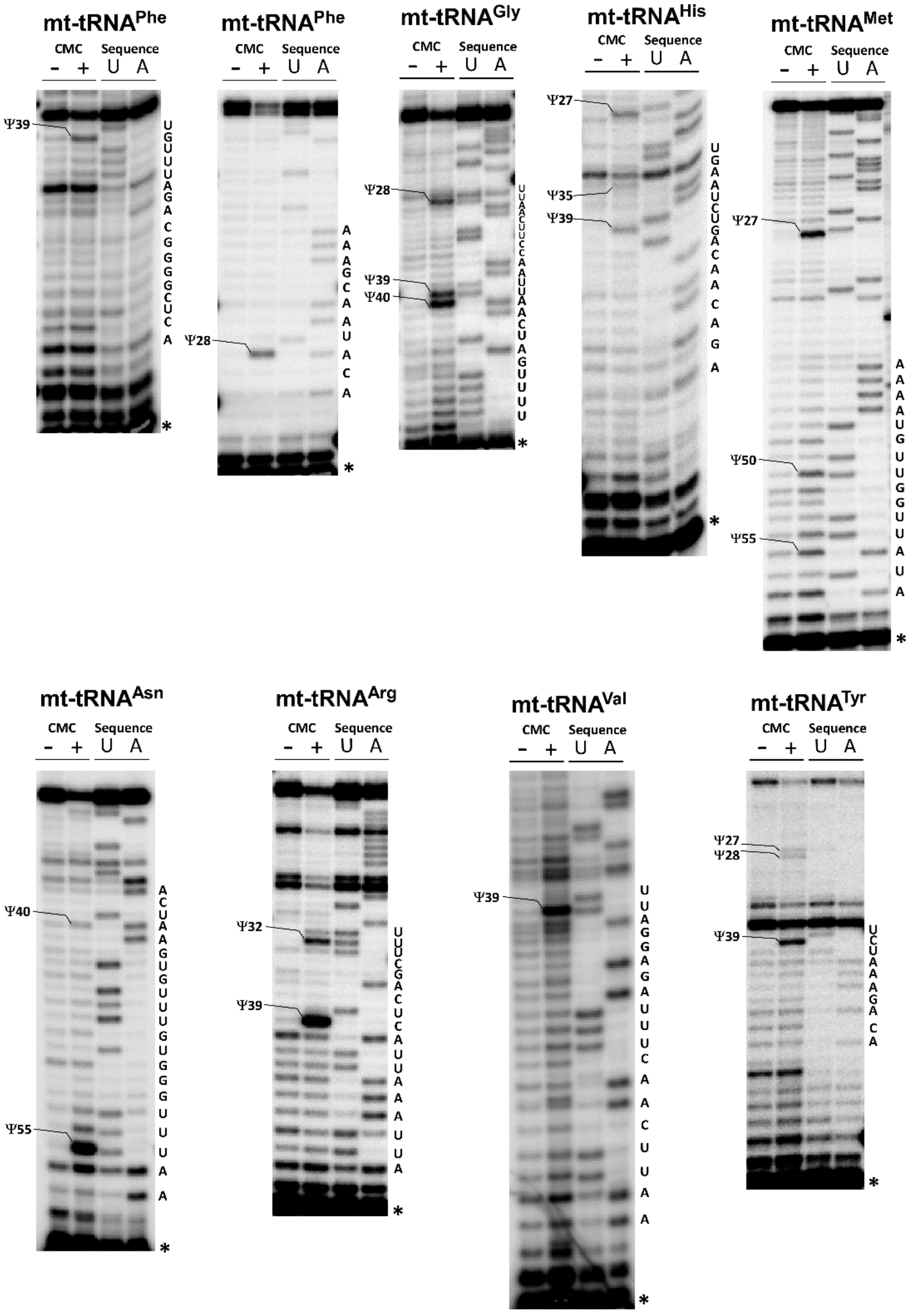

Supplementary Figure 4. CMC-PE analysis for detection of Ψ sites in human mt-tRNAs

HeLa total RNA treated with (+) or without (-) CMC were reverse-transcribed with a primer specific for each human mt-tRNA. The sequence ladders for U and A were generated under the same conditions in the presence of ddATP and ddTTP, respectively. Positions of Ψs and sequence are shown with each gel image. Source data are provided as a Source Data file.

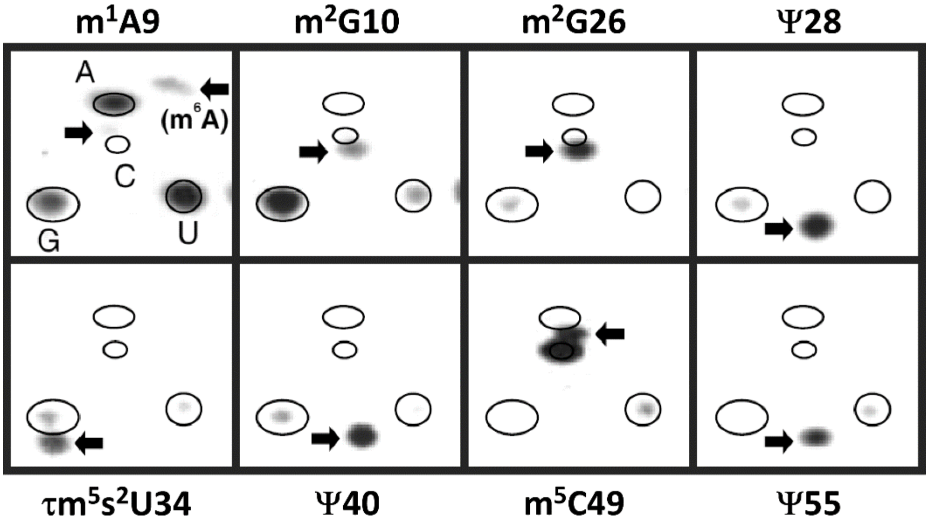

Supplementary Figure 5. Post-transcriptional modifications in human mt-tRNA^{Glu}, determined by the post-labeling method.

Eight modifications in human mt-tRNA^{Glu} were determined by 2D-TLC. The spot corresponding to each modification is indicated by an arrow. Separation patterns for unmodified nucleotide 5’-monophosphates (A, C, G and U) are indicated by ellipses.

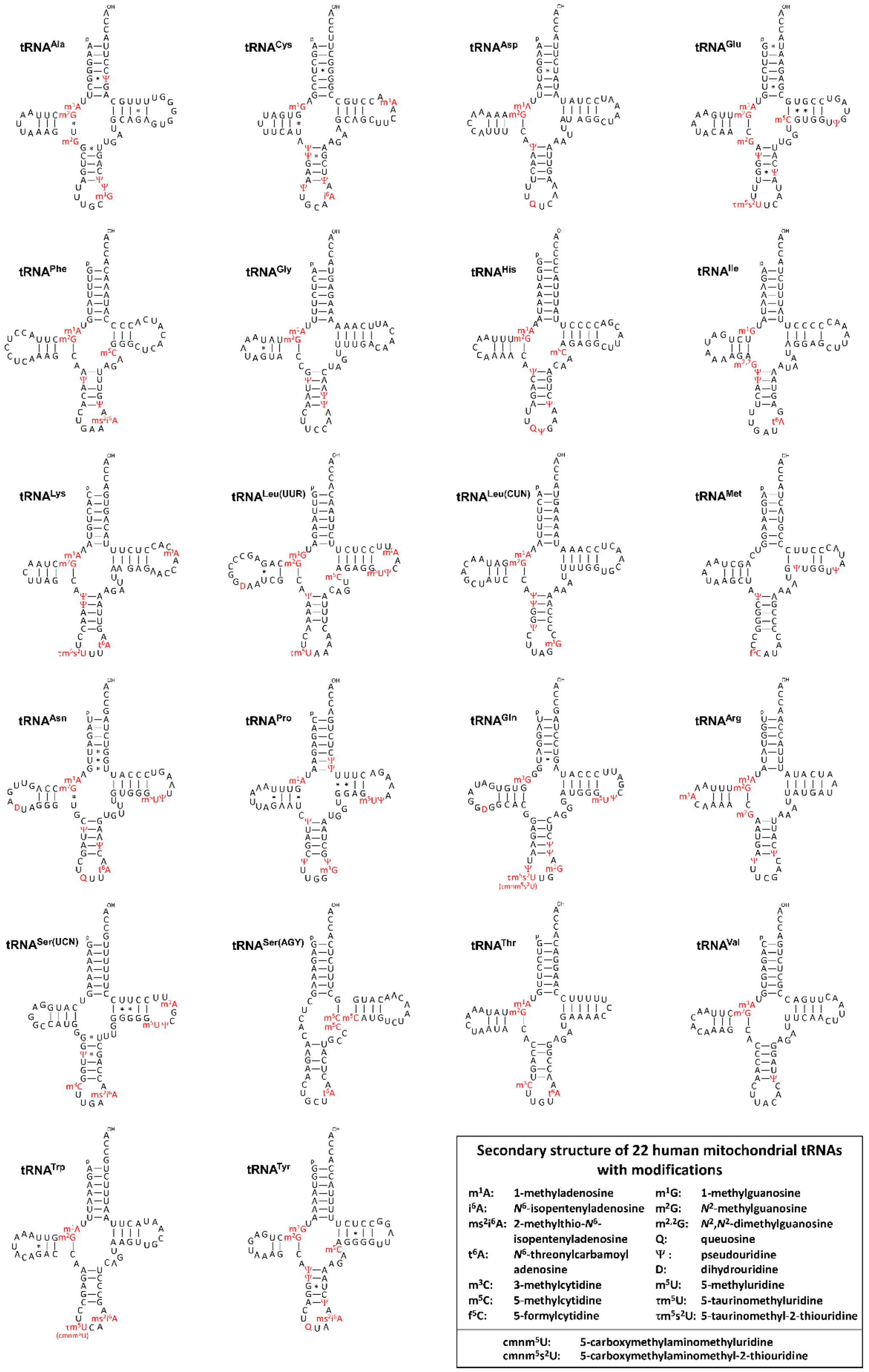

Supplementary Figure 6. Post-transcriptional modifications in all species of human mt-tRNAs.

Supplementary Table 1. List of confirmed and predicted genes responsible for post-transcriptional modifications in human mitochondrial tRNAs

Position [a]	tRNA species	Modification [b]	Confirmed gene(s) in human or mammals	Predicted gene(s) in human	Human diseases
9	Ala, Asp, Glu, Phe, Gly, His, Lys, Leu(CUN), Asn, Pro, Arg, Thr, Val, Trp	m^1A	*MRPP1* and *MRPP2* (formerly known as *TRMT10C* and *SDR5C1*, respectively) [1]		HSD10 disease [2]
9	Cys, Ile, Leu(UUR), Gln, Tyr	m^1G	*MRPP1* and *MRPP2* [1]		HSD10 disease [2]
10	Ala, Asp, Glu, Phe, Gly, His, Lys, Leu(UUR), Leu(CUN), Asn, Arg, Thr, Val, Trp, Tyr	m^2G		*TRMT11* and *TRMT112* [3]	
16	Arg	m^1A		*TRMT61B* [4,5]	
20	Leu(UUR), Asn, Gln	D		*DUS2* [6]	
26	Ala, Glu, Arg	m^2G	*TRMT1* [7]		Intellectual disability [8,9]
26	Ile	$m^{2,2}G$	*TRMT1* [7,10,11]		Intellectual disability [8,9]
27	Cys, Asp, His, Ile, Lys, Leu(UUR), Leu(CUN), Met, Pro, Tyr	Ψ	*PUS1* [12]		MLASA [13]
28	Cys, Glu, Phe, Gly, Ile, Lys, Leu(CUN), Asn, Ser(UCN), Tyr	Ψ	*PUS1* [12]		MLASA [13]
31	Leu(CUN)	Ψ		*RPUSD1, 2, 3 or 4*	
32	Cys, Pro, Arg	Ψ		*RPUSD1, 2, 3 or 4*	

32	Ser(UCN), Thr	m^{3}C		*METTL2A*, *2B*, *6* or *8* [14-16]	
33	Gln	Ψ		*RPUSD1, 2, 3 or 4*	
34	Leu(UUR), Trp, (Glu, Lys, Gln)	τm^{5}U	*GTPBP3* and *MTO1* [17,18]		MELAS (lack of τm^{5}U in mutant tRNA$^{Leu(UUR)}$) [19,20]<br>Hypertrophic cardiomyopathy and lactic acidosis and encephalopathy, Leigh syndrome [21,22] Hypertrophic cardiomyopathy and lactic acidosis [23-25]
34	Glu, Lys, Gln	τm^{5}s^{2}U [c]	*MTU1* [c] [26,27] and	*NFS1* [c] [28]	MERRF (lack of τm^{5}s^{2}U in mutant tRNA^{Lys}) [29]<br>RILF [30-33]
34	Met	f^{5}C	*NSUN3* [34-36] and *ALKBH1* [36,37]		Combined mitochondrial respiratory chain complex deficiency [35]
34	Asp, His, Asn, Tyr	Q	*QTRT1* and *QTRT2* (This work)		
35	His	Ψ		*PUS7* [38]	
37	Ile, Lys, Asn, Ser(AGY), Thr	t^{6}A	*YRDC* and *OSGEPL1* [39]		
37	Cys, (Phe, Ser(UCN), Trp, Tyr)	i^{6}A	*TRIT1* [40]		Encephalopathy and myoclonic epilepsy with multiple OXPHOS deficiencies [41].
37	Phe, Ser(UCN), Trp, Tyr	ms^{2}i^{6}A [d]	*CDK5RAP1* **[d]** [42,43]		
37	Ala, Leu(CUN), Pro, Gln	m^{1}G	*TRMT5* [44,45]		Multiple mitochondrial respiratory chain complex deficiencies [45]
38	Ala, Pro	Ψ		*PUS3* [46]	
39	Ala, Cys, Phe, Gly,	Ψ	*RPUSD4* [e] [47]		

	His, Gln, Arg, Val, Tyr				
40	Glu, Gly, Asn, Gln	Ψ		*PUS3*	
48	Phe, His, Leu(UUR), Ser(AGY), Tyr	m^5C	*NSUN2* [48,49]		Intellectual disability [50-52]
49	Glu, Ser(AGY)	m^5C	*NSUN2* [48,49]		Intellectual disability [50-52]
50	Ser(AGY)	m^5C	*NSUN2* [48,49]		Intellectual disability [50-52]
50	Met	Ψ		*RPUSD4* [47]	
54	Leu(UUR), Asn, Pro, Gln, Ser(UCN)	m^5U	*TRMT2B* [53,54]		
55	Glu, Leu(UUR), Met, Asn, Pro, Gln, Ser(UCN)	Ψ		*TRUB2* [55]	
58	Cys, Lys, Leu(UUR), Ser(UCN)	m^1A	*TRMT61B* [4]		
66	Pro	Ψ		*PUS1*	
67	Pro	Ψ		*PUS1* [56]	
68	Ala	Ψ		*PUS1*	

a: The numbering system for tRNA refers to the tRNAdb compilation [57].

b: Symbols for modifications originate from MODOMICS (http://modomics.genesilico.pl/) [58].

c: MTU1 and NFS1 are involved in 2-thiolation of τm^5s^2U34. *MTU1* (Mitochondrial tRNA-specific 2-thiouridylase 1) is also known as *TRMU*; the name originated from bacterial *trmU* (tRNA methyltransferase U). However, *trmU* (renamed *mnmA*) was mis-annotated, as it is not a tRNA methyltransferase. **d**: CDK5RAP1 is required for 2-methylthiolation of ms^2i^6A37 following 6-isopentenylation of A37. **e**: The literature [47] described that Ψ39 in $tRNA^{Gly}$ was not affected by knockdown of the gene.

MLASA, mitochondrial myopathy and dideroblastic anemia. MELAS, mitochondrial myopathy, encephalopathy, lactic acidosis and stroke-like episodes. MERRF, myoclonus epilepsy associated with ragged-red fibers. RILF, reversible infantile liver failure.

References for Supplemental information

1. Vilardo, E. et al. A subcomplex of human mitochondrial RNase P is a bifunctional methyltransferase--extensive moonlighting in mitochondrial tRNA biogenesis. *Nucleic Acids Res* **40**, 11583-93 (2012).
2. Zschocke, J. HSD10 disease: clinical consequences of mutations in the HSD17B10 gene. *J Inherit Metab Dis* **35**, 81-9 (2012).
3. Purushothaman, S.K., Bujnicki, J.M., Grosjean, H. & Lapeyre, B. Trm11p and Trm112p are both required for the formation of 2-methylguanosine at position 10 in yeast tRNA. *Mol Cell Biol* **25**, 4359-70 (2005).
4. Chujo, T. & Suzuki, T. Trmt61B is a methyltransferase responsible for 1-methyladenosine at position 58 of human mitochondrial tRNAs. *RNA* **18**, 2269-76 (2012).
5. Bar-Yaacov, D. et al. Mitochondrial 16S rRNA Is Methylated by tRNA Methyltransferase TRMT61B in All Vertebrates. *PLoS Biol* **14**, e1002557 (2016).
6. Xing, F., Hiley, S.L., Hughes, T.R. & Phizicky, E.M. The specificities of four yeast dihydrouridine synthases for cytoplasmic tRNAs. *J Biol Chem* **279**, 17850-60 (2004).
7. Dewe, J.M., Fuller, B.L., Lentini, J.M., Kellner, S.M. & Fu, D. TRMT1-catalyzed tRNA modifications are required for redox homeostasis to ensure proper cellular proliferation and oxidative stress survival. *Mol Cell Biol* **37**(2017).
8. Najmabadi, H. et al. Deep sequencing reveals 50 novel genes for recessive cognitive disorders. *Nature* **478**, 57-63 (2011).
9. Davarniya, B. et al. The Role of a Novel TRMT1 Gene Mutation and Rare GRM1 Gene Defect in Intellectual Disability in Two Azeri Families. *PLoS One* **10**, e0129631 (2015).
10. Liu, J. & Straby, K.B. The human tRNA(m(2)(2)G(26))dimethyltransferase: functional expression and characterization of a cloned hTRM1 gene. *Nucleic Acids Res* **28**, 3445-51 (2000).
11. Ellis, S.R., Morales, M.J., Li, J.M., Hopper, A.K. & Martin, N.C. Isolation and characterization of the TRM1 locus, a gene essential for the N2,N2-dimethylguanosine modification of both mitochondrial and cytoplasmic tRNA in Saccharomyces cerevisiae. *J Biol Chem* **261**, 9703-9 (1986).
12. Patton, J.R., Bykhovskaya, Y., Mengesha, E., Bertolotto, C. & Fischel-Ghodsian, N. Mitochondrial myopathy and sideroblastic anemia (MLASA): missense mutation in the pseudouridine synthase 1 (PUS1) gene is associated with the loss

of tRNA pseudouridylation. *J Biol Chem* **280**, 19823-8 (2005).
13. Bykhovskaya, Y., Casas, K., Mengesha, E., Inbal, A. & Fischel-Ghodsian, N. Missense mutation in pseudouridine synthase 1 (PUS1) causes mitochondrial myopathy and sideroblastic anemia (MLASA). *Am J Hum Genet* **74**, 1303-8 (2004).
14. Noma, A. et al. Actin-binding protein ABP140 is a methyltransferase for 3-methylcytidine at position 32 of tRNAs in Saccharomyces cerevisiae. *RNA* **17**, 1111-9 (2011).
15. D'Silva, S., Haider, S.J. & Phizicky, E.M. A domain of the actin binding protein Abp140 is the yeast methyltransferase responsible for 3-methylcytidine modification in the tRNA anti-codon loop. *RNA* **17**, 1100-10 (2011).
16. Xu, L. et al. Three distinct 3-methylcytidine (m3C) methyltransferases modify tRNA and mRNA in mice and humans. *J Biol Chem* **292**, 14695-14703 (2017).
17. Asano, K. et al. Metabolic and chemical regulation of tRNA modification associated with taurine deficiency and human disease. *Nucleic Acids Res* **46**, 1565-1583 (2018).
18. Fakruddin, M. et al. Defective Mitochondrial tRNA Taurine Modification Activates Global Proteostress and Leads to Mitochondrial Disease. *Cell Rep* **22**, 482-496 (2018).
19. Yasukawa, T. et al. Modification defect at anticodon wobble nucleotide of mitochondrial tRNAs^{Leu}(UUR) with pathogenic mutations of mitochondrial myopathy, encephalopathy, lactic acidosis, and stroke-like episodes. *J Biol Chem* **275**, 4251-7 (2000).
20. Kirino, Y., Goto, Y., Campos, Y., Arenas, J. & Suzuki, T. Specific correlation between the wobble modification deficiency in mutant tRNAs and the clinical features of a human mitochondrial disease. *Proc Natl Acad Sci U S A* **102**, 7127-32 (2005).
21. Kohda, M. et al. A Comprehensive Genomic Analysis Reveals the Genetic Landscape of Mitochondrial Respiratory Chain Complex Deficiencies. *PLoS Genet* **12**, e1005679 (2016).
22. Kopajtich, R. et al. Mutations in GTPBP3 cause a mitochondrial translation defect associated with hypertrophic cardiomyopathy, lactic acidosis, and encephalopathy. *Am J Hum Genet* **95**, 708-20 (2014).
23. O'Byrne, J.J. et al. The genotypic and phenotypic spectrum of MTO1 deficiency. *Mol Genet Metab* **123**, 28-42 (2018).
24. Baruffini, E. et al. MTO1 mutations are associated with hypertrophic

cardiomyopathy and lactic acidosis and cause respiratory chain deficiency in humans and yeast. *Hum Mutat* **34**, 1501-9 (2013).
25. Ghezzi, D. et al. Mutations of the Mitochondrial-tRNA Modifier MTO1 Cause Hypertrophic Cardiomyopathy and Lactic Acidosis. *Am J Hum Genet* **90**, 1079-87 (2012).
26. Umeda, N. et al. Mitochondria-specific RNA-modifying enzymes responsible for the biosynthesis of the wobble base in mitochondrial tRNAs. Implications for the molecular pathogenesis of human mitochondrial diseases. *J Biol Chem* **280**, 1613-24 (2005).
27. Wu, Y. et al. Mtu1-Mediated Thiouridine Formation of Mitochondrial tRNAs Is Required for Mitochondrial Translation and Is Involved in Reversible Infantile Liver Injury. *PLoS Genet* **12**, e1006355 (2016).
28. Nakai, Y. et al. Yeast Nfs1p is involved in thio-modification of both mitochondrial and cytoplasmic tRNAs. *J Biol Chem* **279**, 12363-8 (2004).
29. Yasukawa, T. et al. Defect in modification at the anticodon wobble nucleotide of mitochondrial $tRNA^{Lys}$ with the MERRF encephalomyopathy pathogenic mutation. *FEBS Lett* **467**, 175-8 (2000).
30. Zeharia, A. et al. Acute infantile liver failure due to mutations in the TRMU gene. *Am J Hum Genet* **85**, 401-7 (2009).
31. Schara, U. et al. Acute liver failure with subsequent cirrhosis as the primary manifestation of TRMU mutations. *J Inherit Metab Dis* **34**, 197-201 (2011).
32. Uusimaa, J. et al. Reversible infantile respiratory chain deficiency is a unique, genetically heterogenous mitochondrial disease. *J Med Genet* **48**, 660-8 (2011).
33. Gaignard, P. et al. Mitochondrial Infantile Liver Disease due to TRMU Gene Mutations: Three New Cases. *JIMD Rep* **11**, 117-23 (2013).
34. Nakano, S. et al. NSUN3 methylase initiates 5-formylcytidine biogenesis in human mitochondrial tRNA(Met). *Nat Chem Biol* **12**, 546-51 (2016).
35. Van Haute, L. et al. Deficient methylation and formylation of mt-tRNA(Met) wobble cytosine in a patient carrying mutations in NSUN3. *Nat Commun* **7**, 12039 (2016).
36. Haag, S. et al. NSUN3 and ABH1 modify the wobble position of mt-$tRNA^{Met}$ to expand codon recognition in mitochondrial translation. *EMBO J* **35**, 2104-2119 (2016).
37. Kawarada, L. et al. ALKBH1 is an RNA dioxygenase responsible for cytoplasmic and mitochondrial tRNA modifications. *Nucleic Acids Res* **45**, 7401-15 (2017).
38. Behm-Ansmant, I. et al. The Saccharomyces cerevisiae U2

snRNA:pseudouridine-synthase Pus7p is a novel multisite-multisubstrate RNA:Psi-synthase also acting on tRNAs. *RNA* **9**, 1371-82 (2003).
39. Lin, H. et al. CO2-sensitive tRNA modification associated with human mitochondrial disease. *Nat Commun* **9**, 1875 (2018).
40. Lamichhane, T.N., Mattijssen, S. & Maraia, R.J. Human Cells Have a Limited Set of tRNA Anticodon Loop Substrates of the tRNA Isopentenyltransferase TRIT1 Tumor Suppressor. *Mol Cell Biol* **33**, 4900-8 (2013).
41. Yarham, J.W. et al. Defective i6A37 modification of mitochondrial and cytosolic tRNAs results from pathogenic mutations in TRIT1 and its substrate tRNA. *PLoS Genet* **10**, e1004424 (2014).
42. Reiter, V. et al. The CDK5 repressor CDK5RAP1 is a methylthiotransferase acting on nuclear and mitochondrial RNA. *Nucleic Acids Res* **40**, 6235-40 (2012).
43. Wei, F.Y. et al. Cdk5rap1-mediated 2-methylthio modification of mitochondrial tRNAs governs protein translation and contributes to myopathy in mice and humans. *Cell Metab* **21**, 428-42 (2015).
44. Brule, H., Elliott, M., Redlak, M., Zehner, Z.E. & Holmes, W.M. Isolation and characterization of the human tRNA-(N1G37) methyltransferase (TRM5) and comparison to the Escherichia coli TrmD protein. *Biochemistry* **43**, 9243-55 (2004).
45. Powell, C.A. et al. TRMT5 Mutations Cause a Defect in Post-transcriptional Modification of Mitochondrial tRNA Associated with Multiple Respiratory-Chain Deficiencies. *Am J Hum Genet* **97**, 319-28 (2015).
46. Lecointe, F. et al. Characterization of yeast protein Deg1 as pseudouridine synthase (Pus3) catalyzing the formation of psi 38 and psi 39 in tRNA anticodon loop. *J Biol Chem* **273**, 1316-23 (1998).
47. Zaganelli, S. et al. The pseudouridine synthase RPUSD4 is an essential component of mitochondrial RNA granules. *J Biol Chem* **292**, 4519-4532 (2017).
48. Shinoda, S. et al. Mammalian NSUN2 introduces 5-methylcytidines into mitochondrial tRNAs. *Nucleic Acids Res* **47**, 8734-8745 (2019).
49. Van Haute, L. et al. NSUN2 introduces 5-methylcytosines in mammalian mitochondrial tRNAs. *Nucleic Acids Res* **47**, 8720-8733 (2019).
50. Martinez, F.J. et al. Whole exome sequencing identifies a splicing mutation in NSUN2 as a cause of a Dubowitz-like syndrome. *J Med Genet* **49**, 380-5 (2012).
51. Abbasi-Moheb, L. et al. Mutations in NSUN2 cause autosomal-recessive intellectual disability. *Am J Hum Genet* **90**, 847-55 (2012).
52. Khan, M.A. et al. Mutation in NSUN2, which encodes an RNA methyltransferase,

causes autosomal-recessive intellectual disability. *Am J Hum Genet* **90**, 856-63 (2012).

53. Laptev, I. et al. Mouse Trmt2B protein is a dual specific mitochondrial metyltransferase responsible for m(5)U formation in both tRNA and rRNA. *RNA Biol* (2019).
54. Powell, C.A. & Minczuk, M. TRMT2B is responsible for both tRNA and rRNA m(5)U-methylation in human mitochondria. *RNA Biol* **17**, 451-462 (2020).
55. Becker, H.F., Motorin, Y., Planta, R.J. & Grosjean, H. The yeast gene YNL292w encodes a pseudouridine synthase (Pus4) catalyzing the formation of psi55 in both mitochondrial and cytoplasmic tRNAs. *Nucleic Acids Res* **25**, 4493-9 (1997).
56. Behm-Ansmant, I. et al. A previously unidentified activity of yeast and mouse RNA:pseudouridine synthases 1 (Pus1p) on tRNAs. *RNA* **12**, 1583-93 (2006).
57. Sprinzl, M. & Vassilenko, K.S. Compilation of tRNA sequences and sequences of tRNA genes. *Nucleic Acids Res* **33**, D139-40 (2005).
58. Machnicka, M.A. et al. MODOMICS: a database of RNA modification pathways--2013 update. *Nucleic Acids Res* **41**, D262-7 (2013).
