# Supplementary material for: Complete chemical structures of human mitochondrial tRNAs
Source: Nat Commun. 2020 Aug 28;11:4269. doi: 10.1038/s41467-020-18068-6 (PMC7455718; doi:10.1038/s41467-020-18068-6)
Supplement: Supplementary file 2 — Reporting Summary [file 41467_2020_18068_MOESM2_ESM.pdf]

nature research

Corresponding author(s): Tsutomu Suzuki

Last updated by author(s): Jun 30, 2020

Reporting Summary

Nature Research wishes to improve the reproducibility of the work that we publish. This form provides structure for consistency and transparency in reporting. For further information on Nature Research policies, see Authors & Referees and the Editorial Policy Checklist.

Statistics

For all statistical analyses, confirm that the following items are present in the figure legend, table legend, main text, or Methods section.

n/a	Confirmed	
☐	☒	The exact sample size (n) for each experimental group/condition, given as a discrete number and unit of measurement
☐	☒	A statement on whether measurements were taken from distinct samples or whether the same sample was measured repeatedly
☐	☒	The statistical test(s) used AND whether they are one- or two-sided<br>*Only common tests should be described solely by name; describe more complex techniques in the Methods section.*
☒	☐	A description of all covariates tested
☒	☐	A description of any assumptions or corrections, such as tests of normality and adjustment for multiple comparisons
☐	☒	A full description of the statistical parameters including central tendency (e.g. means) or other basic estimates (e.g. regression coefficient) AND variation (e.g. standard deviation) or associated estimates of uncertainty (e.g. confidence intervals)
☐	☒	For null hypothesis testing, the test statistic (e.g. F, t, r) with confidence intervals, effect sizes, degrees of freedom and P value noted<br>*Give P values as exact values whenever suitable.*
☒	☐	For Bayesian analysis, information on the choice of priors and Markov chain Monte Carlo settings
☒	☐	For hierarchical and complex designs, identification of the appropriate level for tests and full reporting of outcomes
☒	☐	Estimates of effect sizes (e.g. Cohen's d, Pearson's r), indicating how they were calculated

Our web collection on statistics for biologists contains articles on many of the points above.

Software and code

Policy information about availability of computer code

Data collection	RNA-Seq raw data was obtained by HiSeq 4000 (100-bp, paired-end and 50-bp, single-end).<br>Gel images were obtained by FLA-7000 and FAS-III.<br>Mass spec data was obtained by LTQ Orbitrap XL, Q Exactive and LCQ duo.<br>Fluorescence image was obtained by AF6500 system.<br>NCBI, UniProt and MITOMAP: public databases.
Data analysis	Qual Browser bundled in Xcalubur 4.3 was used for mass spectrometric analysis.<br>Fiji (ImageJ 1.52q) for graphical analysis.<br>R 3.6.3 for statistical analysis.<br>fastp v0.20.0, Bowtie2 v2.3.3.1, IGV v2.7.2 and STAR 2.7.0a for Illumina-read analysis.<br>Reads were analyzed on Python 3.5.6 with libraries Pysam 0.15.2 and Biopython 1.72.<br>Source codes for analysis of ribosome profiling data were at GitHub (https://github.com/ingolia-lab/RiboSeq).<br>The custom scripts used for tRNA-Ψ-seq are available from the corresponding author upon reasonable request.

For manuscripts utilizing custom algorithms or software that are central to the research but not yet described in published literature, software must be made available to editors/reviewers. We strongly encourage code deposition in a community repository (e.g. GitHub). See the Nature Research guidelines for submitting code & software for further information.

Data

Policy information about availability of data

All manuscripts must include a data availability statement. This statement should provide the following information, where applicable:

- Accession codes, unique identifiers, or web links for publicly available datasets
- A list of figures that have associated raw data
- A description of any restrictions on data availability

Data for tRNA-Ψ-seq and ribosome profiling generated in this study have been deposited in NCBI SRA (PRJNA638467) and GEO (GSE150439), respectively. The tRNA

sequences including modifications have been deposited in DDBJ with the accession codes LC530712, LC530713, LC530714, LC530715, LC530716, LC530717, LC530718, LC530719, LC530720, LC530721, LC530722, LC530723, LC530724, LC530725 , LC530726 for mt-tRNAs for Ala, Cys, Glu, Phe, Gly, His, Met, Asn, Pro, Gln, Arg, Thr, Val, Trp, Tyr, respectively.
The data supporting the findings in this study are available from the corresponding author upon reasonable request.

Field-specific reporting

Please select the one below that is the best fit for your research. If you are not sure, read the appropriate sections before making your selection.

☒ Life sciences ☐ Behavioural & social sciences ☐ Ecological, evolutionary & environmental sciences

For a reference copy of the document with all sections, see nature.com/documents/nr-reporting-summary-flat.pdf

Life sciences study design

All studies must disclose on these points even when the disclosure is negative.

Sample size	No statistical methods were used to predetermine sample size. For sequencing analyses, sample size was two independent biological replicates based on standards in the field. For aggregation assay, sample size was 10 or more to apply Wilcoxon rank sum test.
Data exclusions	No data were excluded.
Replication	Reproducibility for RNA-Seq data and analyses were confirmed by two independent biological replicates. Attempts at replication were successful in aggregation assay.
Randomization	Not applicable because there is no statistic test that requires randomization of samples.
Blinding	Not applicable because blinding would not increase reliability of experiments in this study.

Reporting for specific materials, systems and methods

We require information from authors about some types of materials, experimental systems and methods used in many studies. Here, indicate whether each material, system or method listed is relevant to your study. If you are not sure if a list item applies to your research, read the appropriate section before selecting a response.

Materials & experimental systems

n/a	Involved in the study	
☒	☐	Antibodies
☐	☒	Eukaryotic cell lines
☒	☐	Palaeontology
☒	☐	Animals and other organisms
☒	☐	Human research participants
☒	☐	Clinical data

Methods

n/a	Involved in the study	
☒	☐	ChIP-seq
☒	☐	Flow cytometry
☒	☐	MRI-based neuroimaging

Eukaryotic cell lines

Policy information about cell lines

Cell line source(s)	HEK293T (CRL-11268) and HeLa (CCL-2) cell lines were used. QTRT1 or QTRT2 KO cell lines derived from the HEK293T.
Authentication	None of the cell lines were authenticated.
Mycoplasma contamination	All tested original cell lines showed negative for mycoplasma contamination.
Commonly misidentified lines (See ICLAC register)	No commonly misidentified lines was used in our research.
