# Supplementary material for: Complete chemical structures of human mitochondrial tRNAs
Source: Nat Commun. 2020 Aug 28;11:4269. doi: 10.1038/s41467-020-18068-6 (PMC7455718; doi:10.1038/s41467-020-18068-6)
Supplement: Supplementary file 3 — Description of Additional Supplementary Files [file 41467_2020_18068_MOESM3_ESM.pdf]

Description of Additional Supplementary Files

File Name: Supplementary Data 1
Description: List of RNase T_1- or A-digested fragments of human mt-tRNAs
Representative m/z values we observed are listed.
5'- or 3'-terninal fragments and modification rate are indecated in the Misc. column.
Modification rate was calculated from the signal intensities of observed m/z.
n.d. represents not determined.
Ψs are represented in each fragment according to Figure 4.
Measurement of mt tRNA Ser(AGY) with RNaseA was not performed.
a: These fragments originated from polymorphism in tRNA sequences. In the U-possesing one, no m^5C was observed.
b, e: These fragments were generated by unmodified G due to partial substutution of Q.
c: This fragment was generated by unmodified G due to partial m^1G.
d: Although τm^5s^2U and its derivatives are considered to be susceptive to RNase A digestion, they might be resistant to some extent, which causes partial digestion to produce these fragments.

File Name: Supplementary Data 2
Description: Frequency of mt-tRNA modification
* Average value (%) of RNase T1- and RNase A-digested fragments

File Name: Supplementary Data 3
Description: Mapping Ψs in human mt-tRNAs

File Name: Supplementary Data 4
Description: Primary sequences of 22 species of human mt-tRNAs with post-transcriptional modifications
For symbols indicating modification, see below box.

File Name: Supplementary Data 5
Description: Coincidence of pathogenic mutations and the sites of tRNA modifications in mtDNA

File Name: Supplementary Data 6
Description: List of probes and primers used in this study.

File Name: Supplementary Data 7
Description: Sequence statistics
